# Supplementary material for: Splice-Junction-Based Mapping of Alternative Isoforms in the Human Proteome
Source: Cell Rep. Author manuscript; Available in PMC 2020 Jan 15. (PMC6961840; doi:10.1016/j.celrep.2019.11.026)
Supplement: 2 [file NIHMS1546469-supplement-2.zip › DF1/PXD009737/TestisTrypsin/TestisTrypsin_40_PPP1R12A_ISEMEEELKR.pdf]

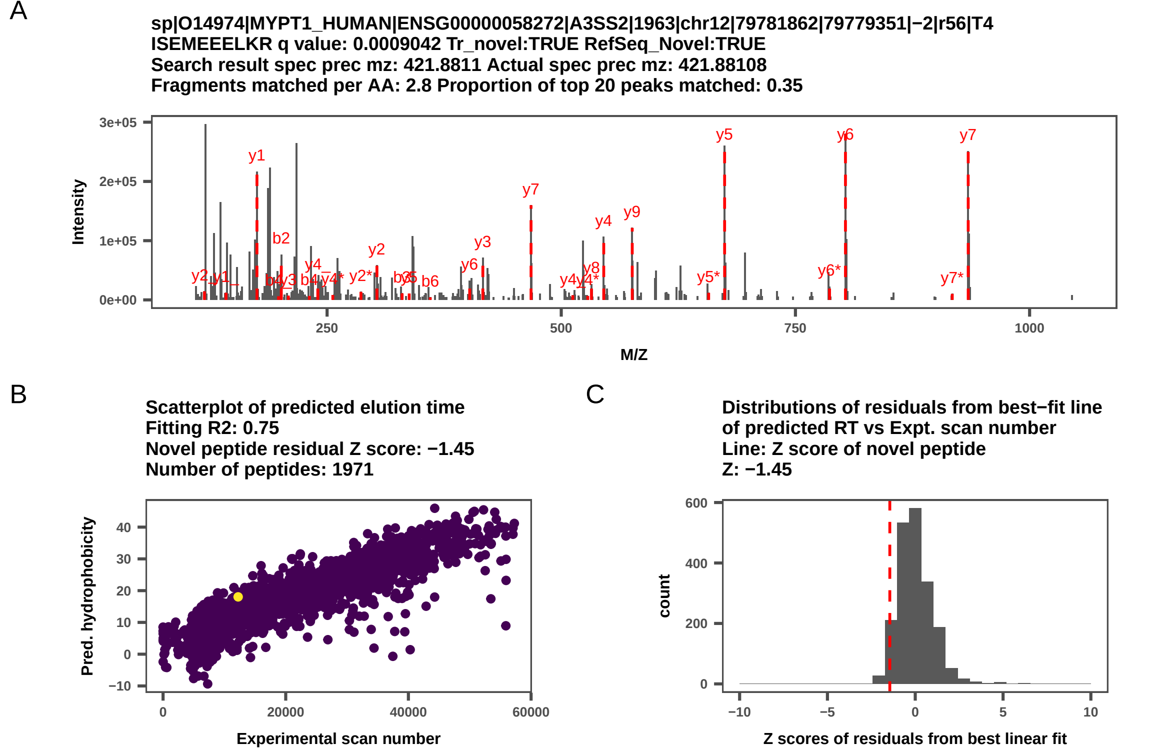
A
sp|O14974|MYPT1_HUMAN|ENSG00000058272|A3SS2|1963|chr12|79781862|79779351|−2|r56|T4
ISEMEEELKR q value: 0.0009042 Tr_novel:TRUE RefSeq_Novel:TRUE
Search result spec prec mz: 421.8811 Actual spec prec mz: 421.88108
Fragments matched per AA: 2.8 Proportion of top 20 peaks matched: 0.35
Intensity
3e+05
2e+05
1e+05
0e+00
y1
b2
y2
y3
y6
y7
y4
y9
y8
y5
y6
y7
y5*
y6*
y7*
y2*
b6
M/Z
250
500
750
1000
B
Scatterplot of predicted elution time
Fitting R2: 0.75
Novel peptide residual Z score: −1.45
Number of peptides: 1971
Pred. hydrophobicity
40
30
20
10
0
−10
0
20000
40000
60000
Experimental scan number
C
Distributions of residuals from best−fit line
of predicted RT vs Expt. scan number
Line: Z score of novel peptide
Z: −1.45
count
600
400
200
0
−10
−5
0
5
10
Z scores of residuals from best linear fit
